# Supplementary material for: Metal toxin threat in wildland fires determined by geology and fire severity
Source: Nat Commun. 2023 Dec 12;14:8007. doi: 10.1038/s41467-023-43101-9 (PMC10716285; doi:10.1038/s41467-023-43101-9)
Supplement: Supplementary file 1 — Supplementary Information [file 41467_2023_43101_MOESM1_ESM.pdf]

Metal Toxin Threat in Wildland Fires Determined by Geology and Fire Severity

Supplementary Information

Alandra Marie Lopez[1], Juan Lezama Pacheco[1], and Scott Fendorf[1]*

[1] Earth System Science Department, Stanford University, Stanford, CA, 94305, USA

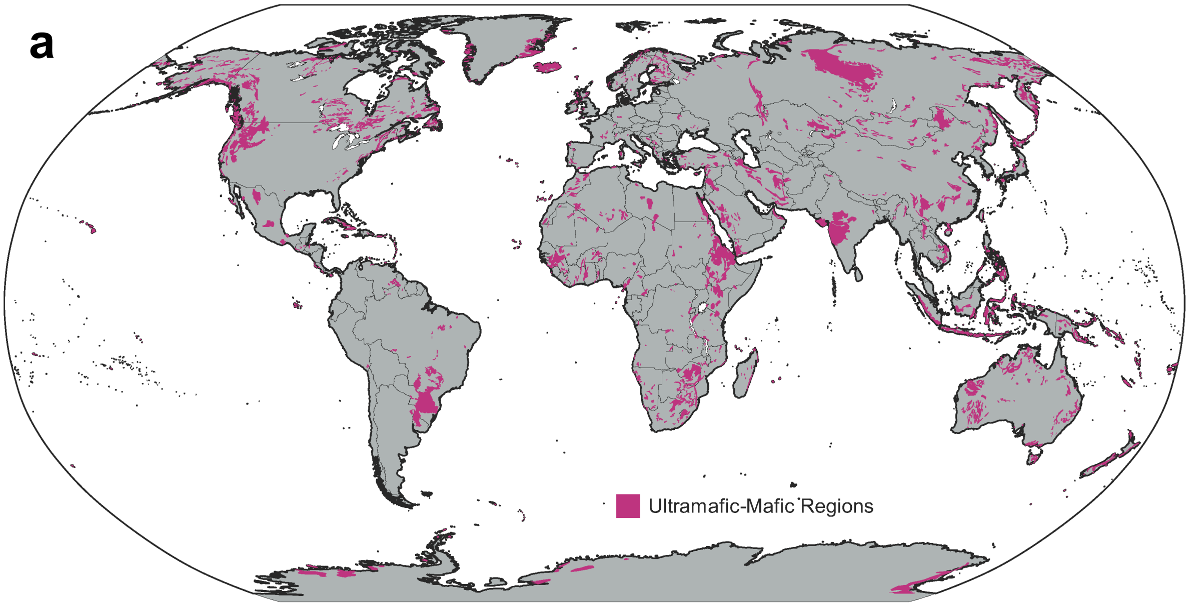

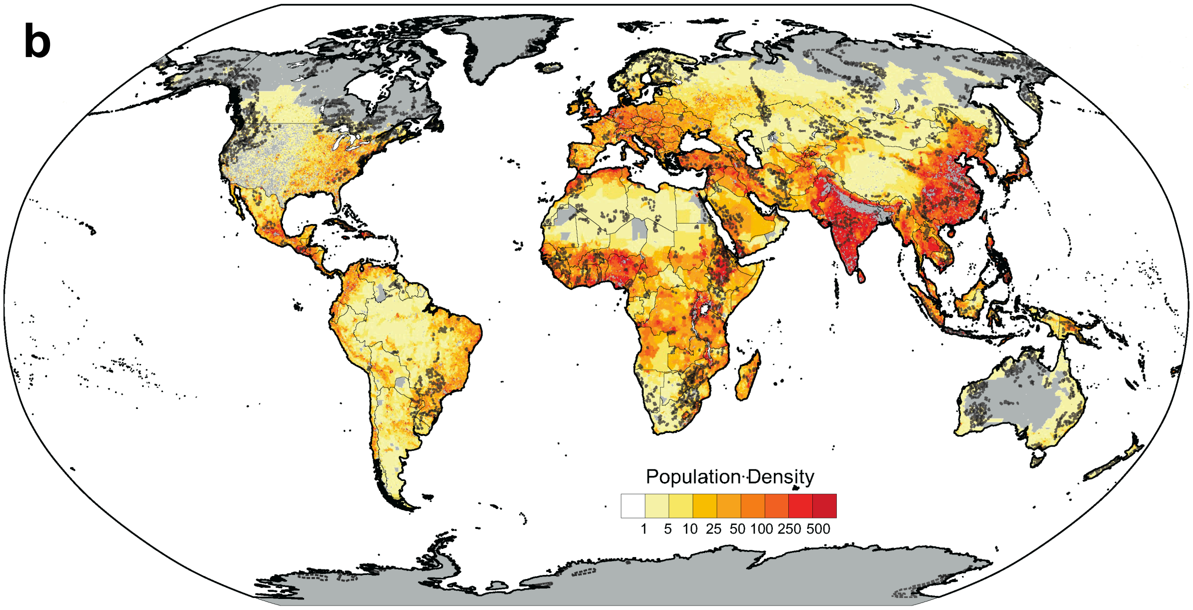

Supplementary Figure 1: **Global maps of metal-rich geology and population density.** a. Global map showing generalized domains of mafic and/or ultramafic geologies, inclusive of their metamorphic equivalents. b. Global map showing total population density using a projection model for 2020 based on a "middle-of-the-road" socio-economic scenario (SSP2) with generalized mafic and ultramafic regions shown with dashed lines.

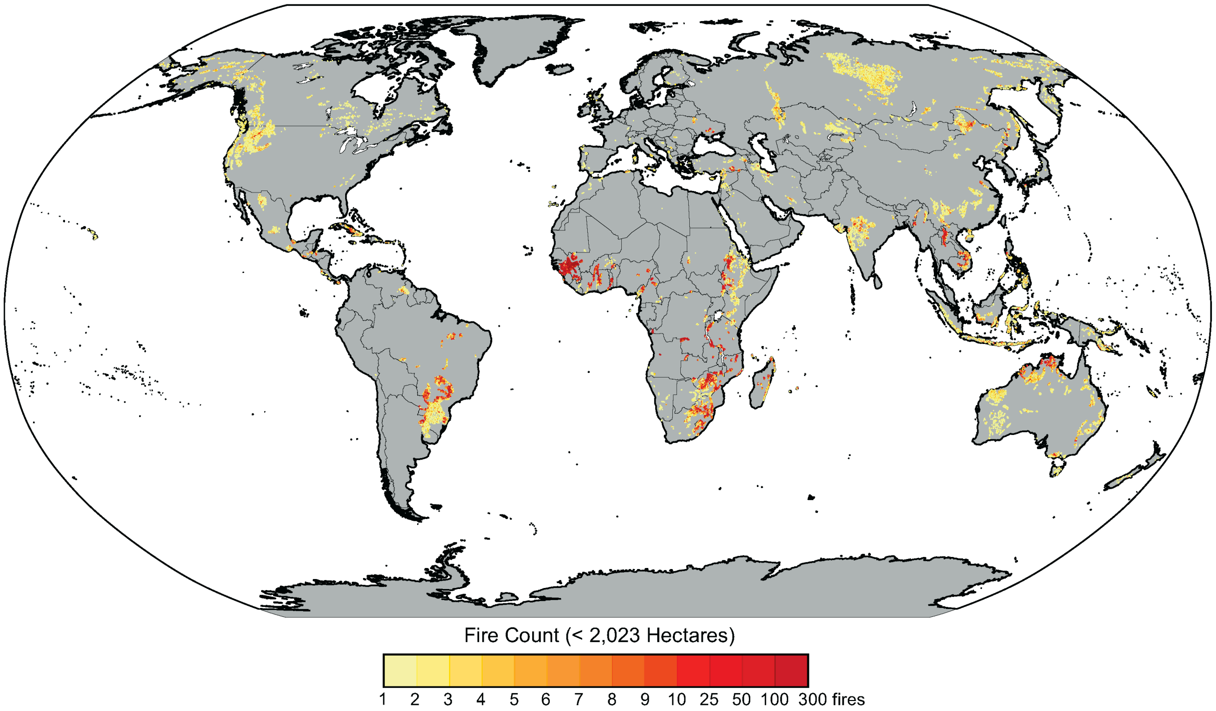

Supplementary Figure 2: **Frequency of small fires in metal-rich landscapes.** Satellite-derived number of fire occurrences less than 2,023 ha within generalized mafic and ultramafic landscapes (Supplementary Figure 1) from 2001-2020.

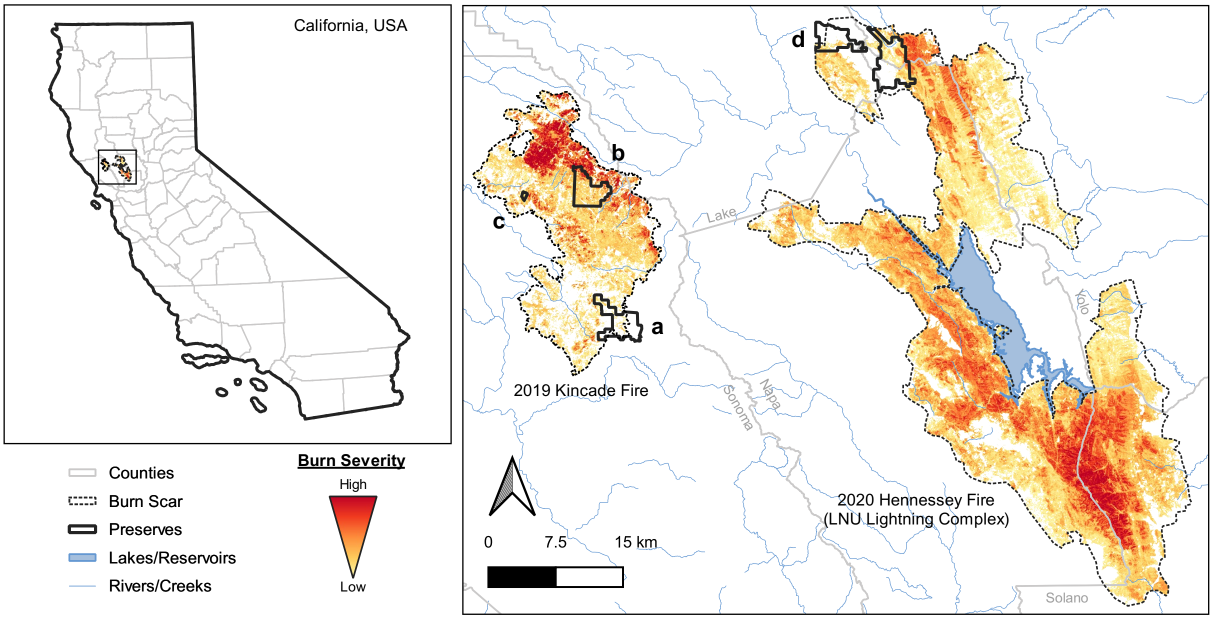

Supplementary Figure 3: **Four natural preserves within burn scars of the 2019 Kincade Fire and 2020 Hennessey Fire.** a. Pepperwood Preserve, b. Modini Preserve, c. White Rock Preserve, and d. McLaughlin Natural Reserve. Visualization of relative burn severity (initial assessment) for each fire was estimated by the absolute difference between pre- and most-fire normalized burn ratio (NBR) using Landsat 8 images and clipped within fire boundaries. For the Kincade Fire, the NBR was calculated based on cloud-free images (LC08_L1TP_045033) from October 1-18, 2019 (pre-fire) and November 2-15, 2019 (post-fire). For the Hennessey Fire (within the LNU Lightning Complex), cloud-free images (LC08_L1TP_044033) used were acquired from August 9-21, 2020 (pre-fire) and August 25-September 5, 2020 (post-fire).

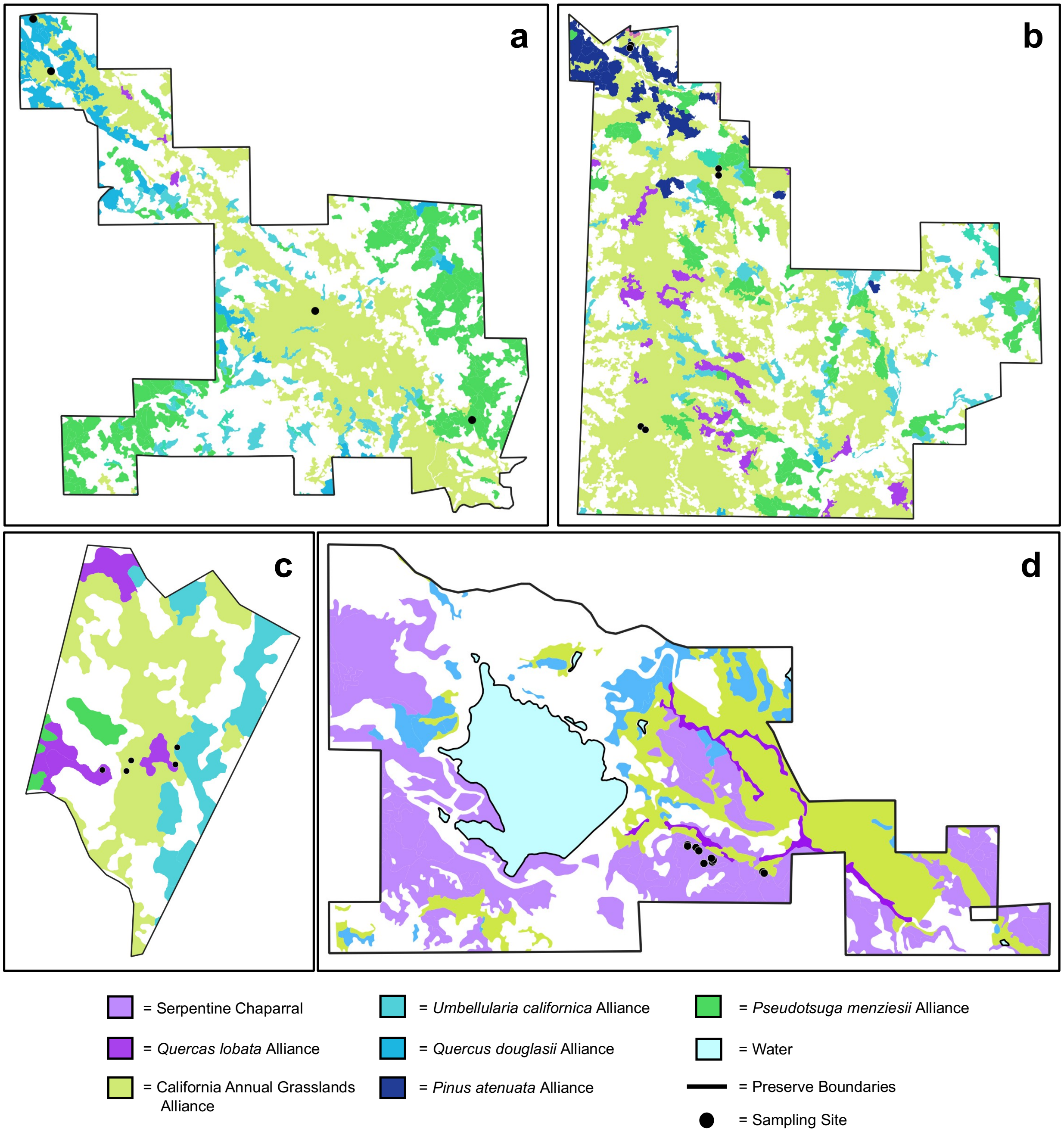

Supplementary Figure 4: **Vegetation classification at each sampling site.** a. Pepperwood Preserve, b. Modini Preserve, c. White Rock Preserve, and d. McLaughlin Natural Reserve. Sampling sites are represented by black points. Additional vegetation classes within regional maps that do not overlap with soil sampling sites were omitted for simplicity.

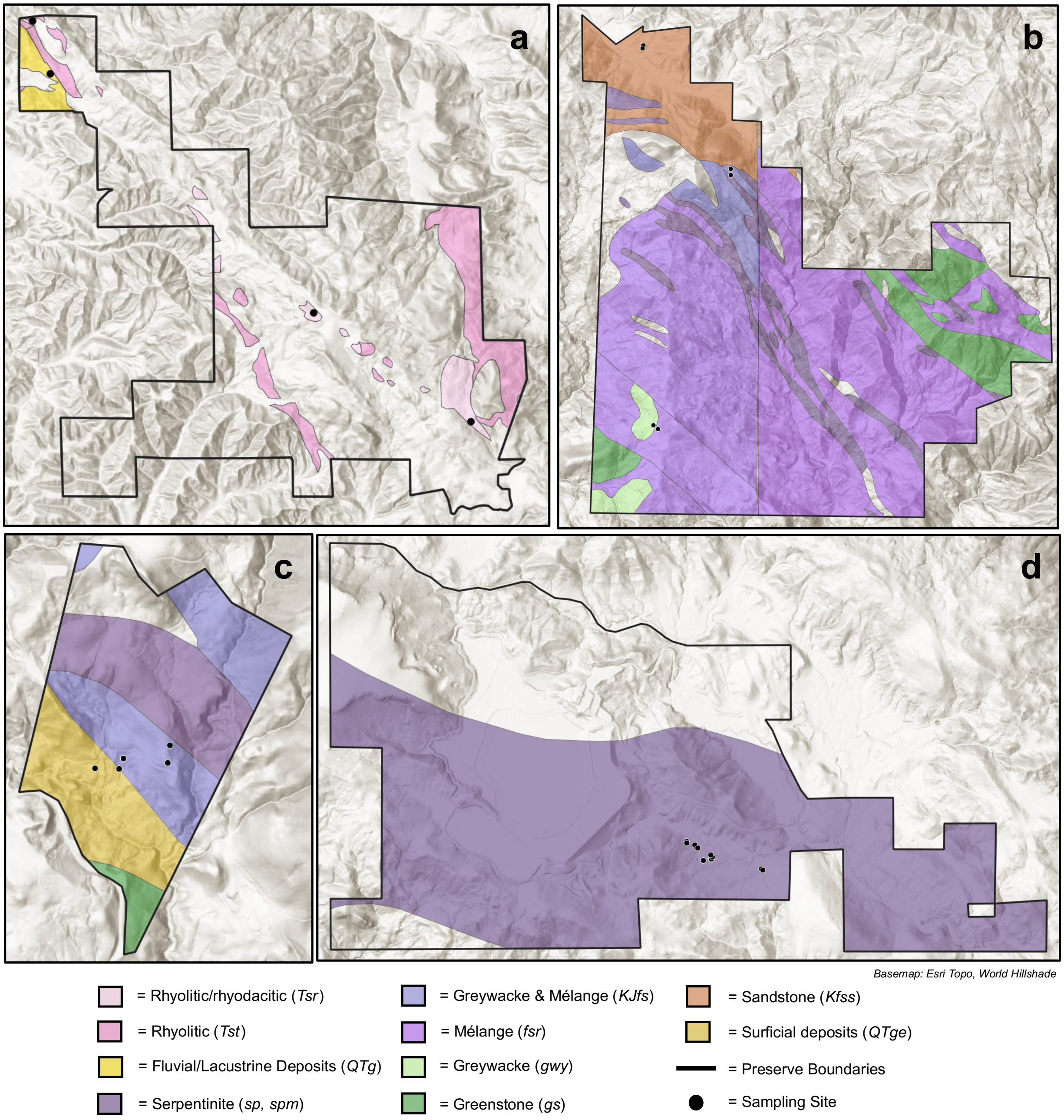

Supplementary Figure 5: **Surface bedrock geology at each sampling site.** a. Pepperwood Preserve, b. Modini Preserve, c. White Rock Preserve, and d. McLaughlin Natural Reserve. Sampling sites are represented by black points. Additional geologic units within regional maps that do not overlap with soil sampling sites were omitted for simplicity.

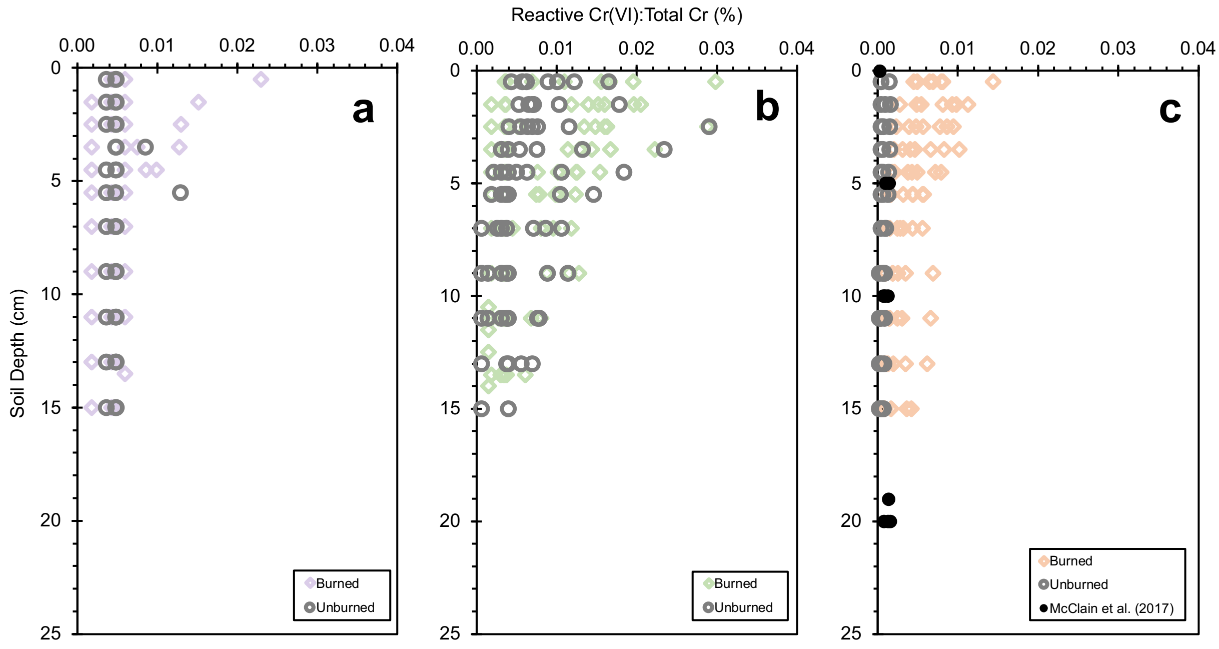

Supplementary Figure 6: **Fraction of total Cr that is reactive Cr(VI) in burned and unburned soils.** The ratio of reactive Cr(VI) to total Cr concentrations (as a percentage) within a. rhyolite-, b. mélange-, c. serpentinite-derived soil profiles (0-16 cm) that were not burned (gray circles; rhyolite, n = 3; mélange, n = 7; serpentinite, n = 3) or were fire-affected (colored diamonds; rhyolite, n = 4, purple; mélange, n = 9, green; serpentinite, n = 7, orange). Percentages were also plotted for serpentinite-derived soil (0-20 cm) from McClain *et al.* (2017) in c. for comparison (black circles) [1]. Each point represents the average percentage for a soil core based on triplicate measurements.

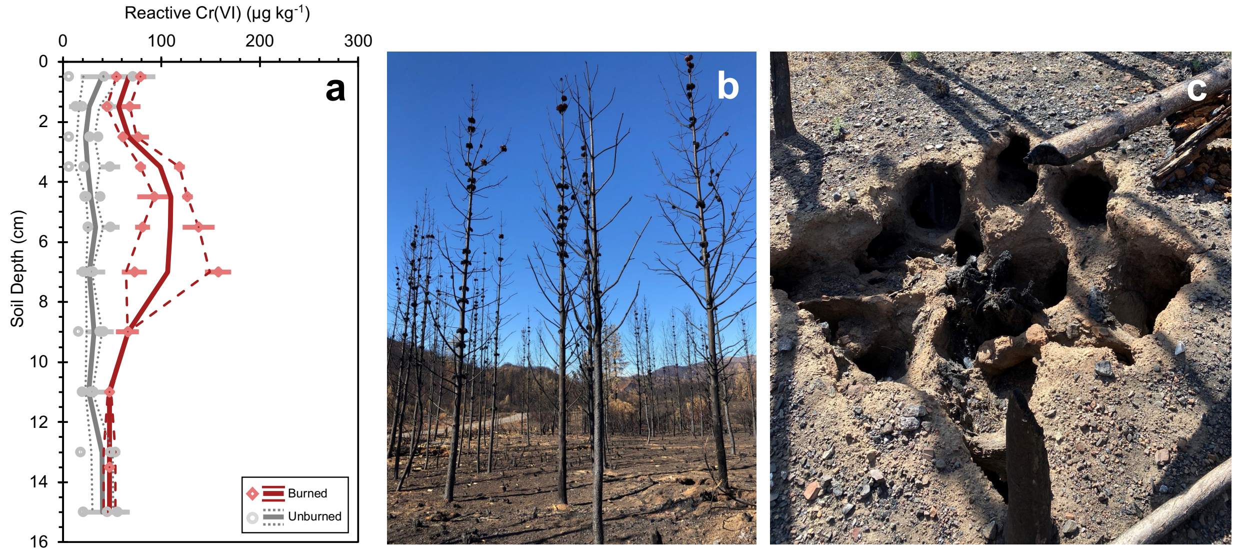

Supplementary Figure 7: **Reactive Cr(VI) concentrations within a severely burned forest.** a. Average reactive Cr(VI) concentrations (µg kg^{-1}) within near surface soil profiles that were considered unburned (gray circles; *n* = 3) and experienced high burn severity (red diamonds; *n* = 2) in a densely vegetated knobcone pine forest. Each point represents the sample average of triplicates and error bars represent the standard error. Solid and dashed lines represent the mean (based on *n* soil cores) and standard error of the means, respectively. b. Photos of the burned forested area immediately after the 2019 Kincade Fire. c. An example of a completely combusted tree root system nearly one year later (September 2020).

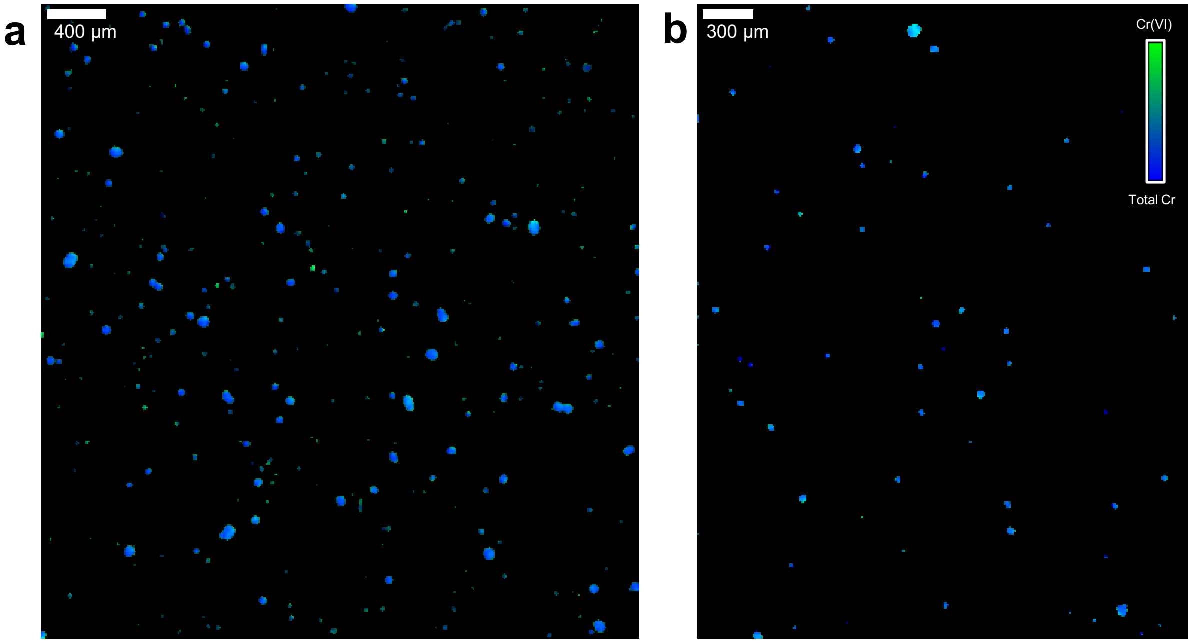

Supplementary Figure 8: **Total Cr(VI) in wind-dispersible soil and ash particles.** μ-XRF image showing particle distribution of total Cr(VI) (green; estimated as the intensity ratio at 5993 and 6010 eV) and total Cr (blue; measured at 6010 eV) within the < 53-μm size fraction of Cr-bearing soil-ash particulates from a. high fire severity site (A7) and b. low fire severity site (A1) in a serpentine chaparral.

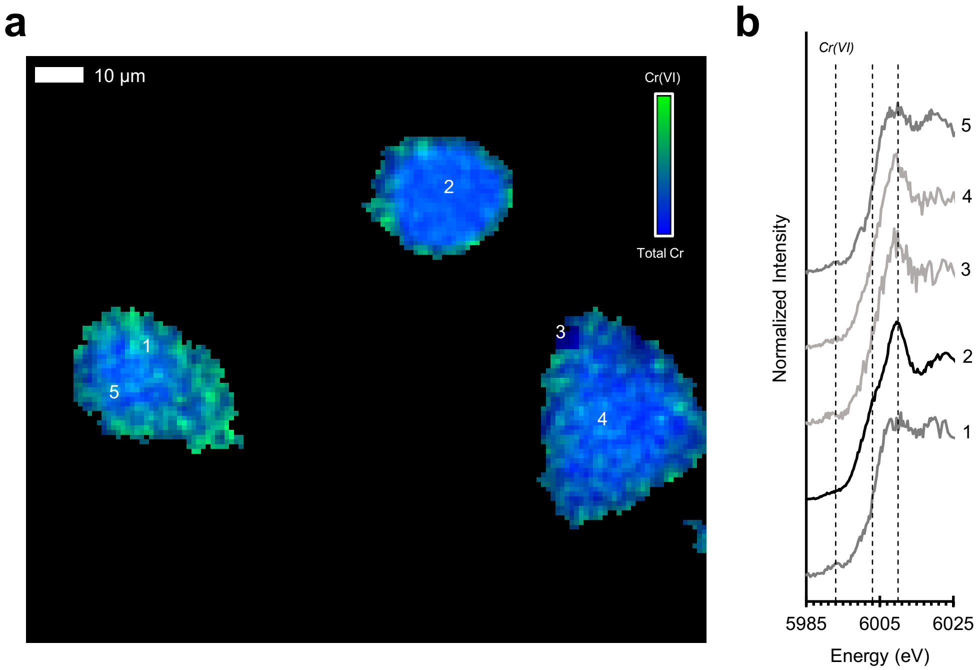

Supplementary Figure 9: **Total Cr(VI) in wind-dispersible soil and ash particles.** a. μ-XRF image (pixel resolution: 1 μm) showing the relative intensity of Cr(VI) (green; estimated as the intensity ratio at 5993 and 6010 eV) and total Cr (blue; measured at 6010 eV) within the < 53-μm size fraction of Cr-bearing soil-ash particulates from a serpentine chaparral that experienced high fire severity (A7). b. Normalized μ-XANES spectra (Cr K-edge) from numbered locations on Cr-bearing particles in a. Dashed lines indicate energies characteristic of Cr(VI) (5993 eV), Cr(III) (6003 eV), and total Cr (6010 eV), at which μ-XRF images were also collected.

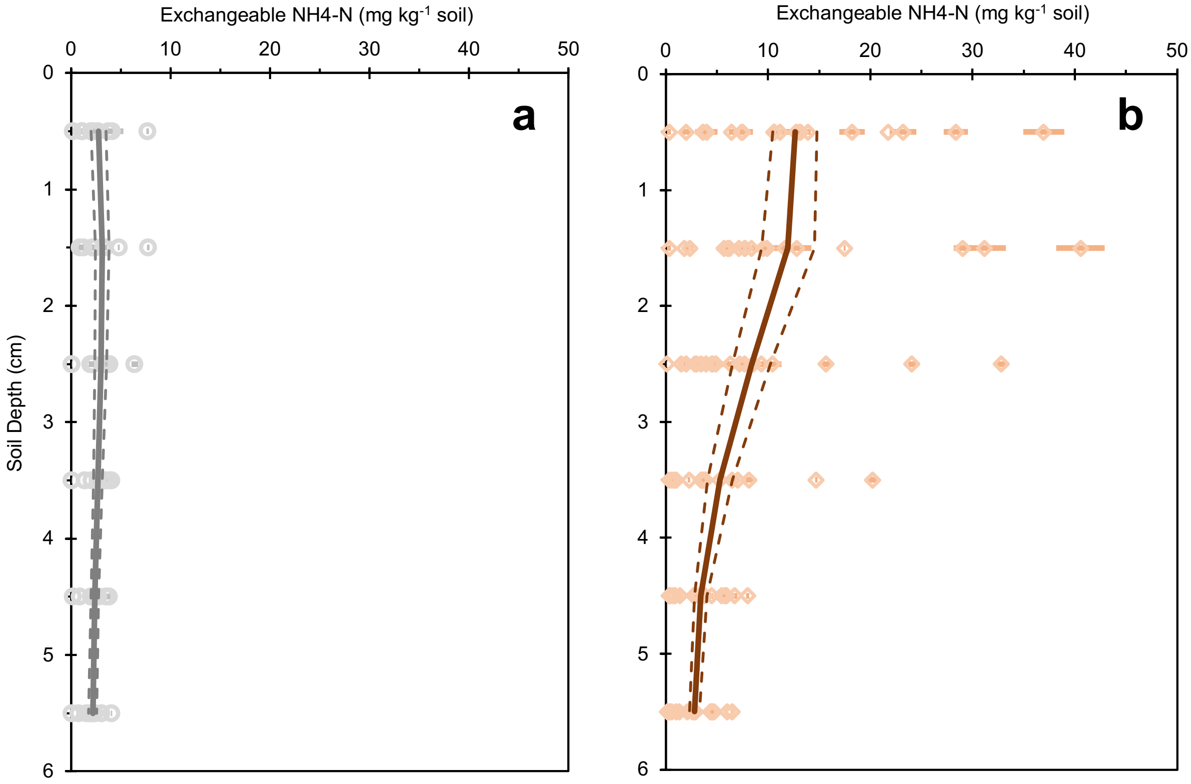

Supplementary Figure 10: **Ammonium concentrations within burned and unburned soils.** Exchangeable NH_4-N concentrations (mg kg^{-1}) across depths (cm) in a. unburned (gray circles, n = 9) soil and b. burned (orange diamonds, n = 19). Each point represents the sample average of triplicates and error bars represent the standard error. The solid and dashed lines represent mean concentrations (based on n soil cores) and standard error of the means, respectively, for burned and unburned soil in the top 6-cm.

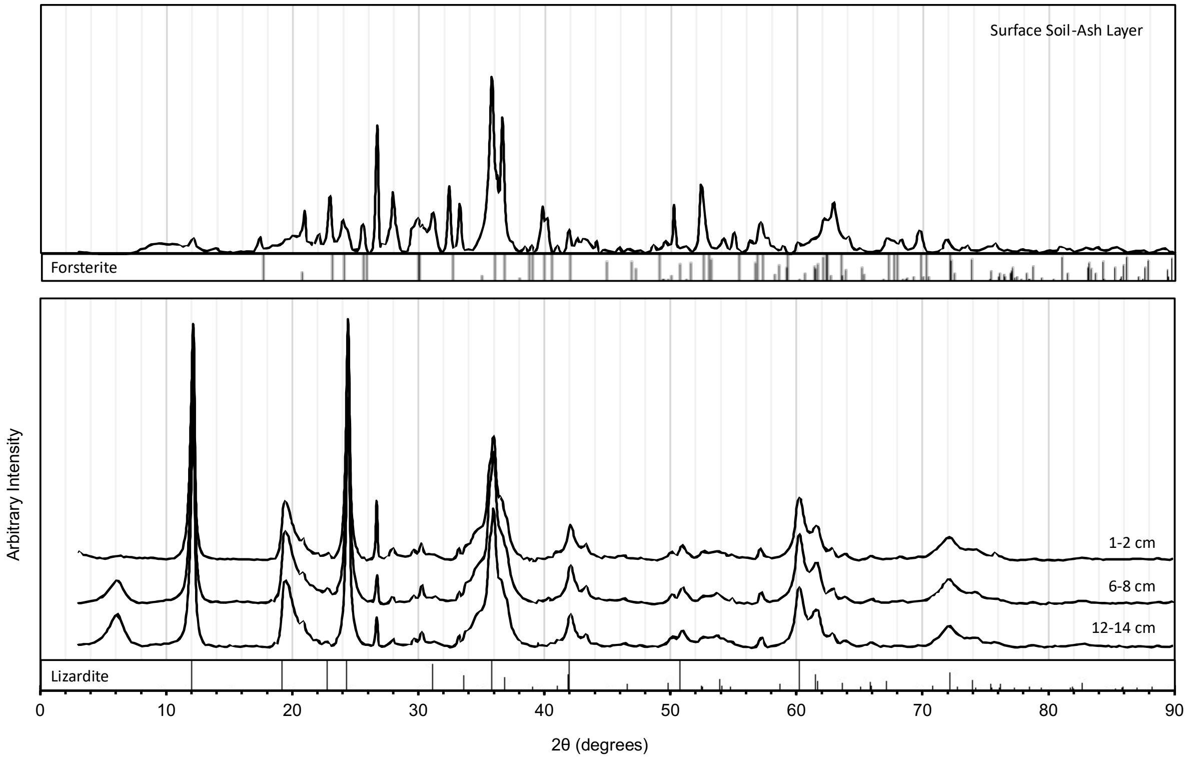

Supplementary Figure 11: **Bulk soil mineral transformations after high fire severity.** X-ray diffraction pattern of bulk mineralogy from a severely burned surface soil ash layer (A7) and associated soil depth intervals (1-2 cm, 6-8 cm, and 12-14 cm) collected from a serpentine chaparral and reference lines for forsterite (PDF Card No. 64741) and lizardite (PDF Card No. 81101).

Supplementary Table 1: Field site descriptions, including preserve location, wildfire, geology, general ecosystem type, estimated fire severity, and average total Cr (mg kg^{-1}) and reactive Cr(VI) concentrations (µg kg^{-1}) for each soil core.

						Total Cr (mg kg^{-1})[d]		Reactive Cr(VI) (µg kg^{-1})[e]			
Soil Core	Preserve[a]	Fire[b]	Geology	Ecosystem	Fire Severity[c]	Mean (0-16 cm)	± SE (0-16 cm)	Mean (0-2 cm)	± SE (0-2 cm)	Mean (10-16 cm)	± SE (10-16 cm)
Fire-Affected Sites											
SC1	MLNR	2020	Serpentinite	Chaparral	L	1137	65	55.54	0.30	23.12	2.51
SC2	MLNR	2020	Serpentinite	Chaparral	L	1711	119	105.96	12.69	19.23	3.61
SC3	MLNR	2020	Serpentinite	Chaparral	L	1595	0	57.57	14.11	13.25	0.39
SC4	MLNR	2020	Serpentinite	Chaparral	M/H	3023	531	220.30	24.89	16.87	2.43
SC5	MLNR	2020	Serpentinite	Chaparral	M/H	2440	296	216.00	23.56	138.15	17.87
SC6	MLNR	2020	Serpentinite	Chaparral	M/H	2003	97	257.33	31.67	67.96	3.31
SC7	MLNR	2020	Serpentinite	Chaparral	M/H	2429	220	211.42	14.21	29.46	3.26
M1	WR	2019	Mélange[f]	Grassland	L	330	32	14.50	8.50	nd	-
M2	WR	2019	Mélange[f]	Grassland	L	404	30	79.02	0.18	nd	-
M3	WR	2019	Mélange[f]	Grassland	L	177	7	32.53	3.75	nd	-
M4	WR	2019	Mélange[f]	Grassland	L	159	5	21.84	2.95	nd	-
M5	WR	2019	Mélange[f]	Forest	L	354	32	57.75	1.19	28.33	0.88
M6	MP	2019	Mélange[f]	Grassland	L	169	15	nd	-	nd	-
M7	MP	2019	Mélange[f]	Grassland	L	289	19	64.94	21.08	19.85	2.40
M8	MP	2019	Mélange[f]	Grassland	L	634	26	83.62	9.35	18.18	0.93
M9	MP	2019	Mélange[f]	Grassland	L	164	3	nd	-	11.09	2.62
F1	PP	2019	Rhyolite	Forest	L	102	7	nd	-	nd	-
F2	PP	2019	Rhyolite	Forest	L	124	2	23.55	4.81	nd	-
F3	PP	2019	Rhyolite	Grassland	L	141	9	nd	-	nd	-
F4	PP	2019	Rhyolite	Grassland	L	338	7	10.81	4.81	nd	-
SF1[g]	MP	2019	Sandstone[h]	Forest	H	686	15	49.49	4.81	-	-
SF2	MP	2019	Sandstone[h]	Forest	H	968	78	73.47	5.51	47.25	0.03
Unburned Sites											
SC8	MLNR		Serpentinite	Chaparral		4070	71	61.04	3.04	32.65	1.42
SC9	MLNR		Serpentinite	Chaparral		3162	253	38.03	7.81	nd	-
SC10	MLNR		Serpentinite	Chaparral		1340	14	nd	-	nd	-
M10	WR		Mélange[f]	Grassland		954	3	45.68	4.41	nd	-
M11	WR		Mélange[f]	Grassland		162	5	27.65	1.06	nd	-
M12	WR		Mélange[f]	Grassland		193	8	16.54	2.90	8.33	2.33
M13	WR		Mélange[f]	Forest		411	35	26.21	2.82	31.33	0.55
M14	MP		Mélange[f]	Grassland		152	10	14.59	3.97	nd	-
M15	MP		Mélange[f]	Grassland		423	47	26.70	0.72	nd	-

M16	MP	Mélange[f]	Grassland	190	8	18.20	1.30	13.98	0.87
F5	PP	Rhyolite	Grassland	125	4	nd	-	nd	-
F6	PP	Rhyolite	Forest	166	16	nd	-	nd	-
F7	PP	Rhyolite	Forest	127	13	nd	-	nd	-
SF3	MP	Sandstone[h]	Forest	1199	63	10.67	3.50	43.99	8.16
SF4	MP	Sandstone[h]	Forest	1081	56	30.74	11.00	19.48	0.78
SF5	MP	Sandstone[h]	Forest	1518	54	58.86	12.08	43.16	6.49

[a] MLNR = McLaughlin Natural Reserve, WR = White Rock Preserve, PP = Pepperwood Preserve, MP = Modini Preserve

[b] 2019 = Kincade Fire, 2020 = Hennessey Fire (LNU Lightning Complex)

[c] L = Low severity, M = Moderate severity, H = High severity

[d] Reported as the mean (n = 3-4 replicate measurements) ± standard error

[e] Reported as the mean (n = 3 replicate measurements for each depth interval) ± standard error of depth means; nd*:* all concentrations within depth range were below limit of detection (12 μg kg^{-1})

[f] Mélange (*KJfs, fsr*) from the Franciscan Complex contains a complex blend of rocks from tectonic mixing, including greenstone, metabasaltic blueschist, greywacke

[g] The soil depth range of core SF1 was 0-10 cm.

[h] Based on chemical analyses, the sandstone-derived soil was consistent with typical mafic chemical properties (geologic unit: *Kfss*)

Supplementary Table 2: Statistical comparison of reactive Cr(VI) concentrations (μg kg^{-1}) in fire-affected (burned) versus unburned soil cores at near surface depths based on geology and fire severity using unpaired, two-sided t tests. Datasets were normal based on the Shapiro-Wilk test, and equal variances were determined using the f-test. Bold, italicized p-values indicate statistically significant differences at the 95% confidence interval (α = 0.05). The number of soil core replicates used to calculate means for each soil type is reported as *n* and detailed in Table S1.

		Fire Affected		Unburned		Difference		Unpaired t-test		
Geology	Fire Severity	*n*	Mean (μg kg^{-1})	*n*	Mean (μg kg^{-1})	Mean (μg kg^{-1})	95% CI (μg kg^{-1})	t-value	df	p-value
Near Surface (0-2 cm)										
Serpentinite	Low	3	73.02	3	35.02	38.00	(-25.69, 101.7)	1.66	4	0.1730
Serpentinite	Mod/High	4	226.3	3	35.02	191.2	(144.3, 238.2)	10.48	5	***0.0001***
Serpentinite	All	7	160.6	3	35.02	125.6	(6.56, 244.6)	2.43	8	***0.0410***
Mélange[a]	Low	9	40.69	7	25.08	15.61	(-9.01, 40.23)	1.41	10	0.1888

[a] Welch's t-test (unequal variances)

Supplementary Table 3: Statistical comparison of reactive Cr(VI) concentrations (μg kg^{-1}) in fire-affected (burned) versus unburned soil cores at deep (control) depths based on geology and fire severity using two-sided Mann-Whitney U tests. One or both datasets were not normally distributed based on the Shapiro-Wilk test. Bold, italicized p-values indicate statistically significant differences at the 95% confidence interval (α = 0.05). The number of soil core replicates used to calculate means for each soil type is reported as *n* and detailed in Table S1.

			Fire Affected		Unburned		Mann-Whitney U Test	
	Geology	Fire Severity	*n*	Mean (μg kg^{-1})	*n*	Mean (μg kg^{-1})	W	p-value
Control Depths (10-16 cm)								
	Serpentinite	Low	3	18.53	3	14.88	6	0.6579
	Serpentinite	Mod/High	4	63.11	3	14.88	10	0.2118
	Serpentinite	All	7	44.01	3	14.88	16	0.2530
	Mélange	Low	9	11.94	7	11.09	33	0.9071

Supplementary Table 4: Statistical comparison of reactive Cr(VI) concentrations (μg kg^{-1}) in near surface versus deep (control) depths in soil cores based on geology and fire severity using two-sided Wilcoxon signed rank test. One or both datasets were not normally distributed based on the Shapiro-Wilk test. Bold, italicized p-values indicate statistically significant differences at the 95% confidence interval (α = 0.05). The number of soil core replicates used to calculate means for each soil type (same for surface and control depths) is reported as *n* and detailed in Table S1.

				0-2 cm	10-16 cm	Wilcox Signed Rank Test	
	Geology	Fire Severity	*n*	Mean (μg kg^{-1})	Mean (μg kg^{-1})	V	p-value
Fire-Affected							
	Serpentinite	All	7	160.6	44.01	28	***0.0156***
	Mélange	Low	9	40.69	11.94	35	***0.0209***
Unburned							
	Serpentinite	-	3	35.02	14.88	3	0.3711
	Mélange	-	7	25.08	11.09	26	***0.0469***

Supplementary Table 5: Statistical comparison of reactive Cr(VI) concentrations (μg kg^{-1}) in near surface versus deep (control) depths in soil cores based on geology and fire severity using two-sided paired t tests. Datasets were normal based on the Shapiro-Wilk test. Bold, italicized p-values indicate statistically significant differences at the 95% confidence interval (α = 0.05). The number of soil core replicates used to calculate means for each soil type (same for surface and control depths) is reported as *n* and detailed in Table S1.

				0-2 cm	10-16 cm	Difference		Paired t-test		
	Geology	Fire Severity	*n*	Mean (μg kg^{-1})	Mean (μg kg^{-1})	Mean (μg kg^{-1})	95% CI (μg kg^{-1})	t-value	df	p-value
Fire-Affected										
	Serpentinite	Low	3	73.02	18.53	54.49	(-16.42,125.4)	3.31	2	0.0806
	Serpentinite	Mod/High	4	226.27	63.11	163.15	(71.56, 254.8)	5.67	3	***0.0109***

Supplementary Table 6: Physicochemical characteristics of bulk soil and ash (up to 2 mm), and selected fine size fractions less than 53 μm, collected from surface layers of the burned serpentine chaparral, and mean elemental concentrations from bulk underlying soil based on geology type (rhyolitic, mélange, and serpentine).

ID	Fire Severity[a]	% Sand[b] (2-0.05 mm)	% Silt[c] (53-2 μm)	% Clay[c] (< 2 μm)	Cr mg kg^{-1}	Fe mg g^{-1}	Mn mg kg^{-1}	Ni mg kg^{-1}	Ca mg g^{-1}	Mg mg g^{-1}	Na mg g^{-1}	K mg g^{-1}
Surface Soil-Ash												
A1	L				1147	64.7	1351	1528	15.5	77.3	4.63	3.76
A2	L				1532	69.1	1203	2530	10.2	158	< 0.1	1.65
A3	M/H				1606	84.2	1480	3117	9.3	148	< 0.1	1.54
A4	M/H	87.5	11.7	0.8	2256	78.9	1438	3380	8.7	159	< 0.1	0.90
A5	M/H				999	57.7	1022	1849	10.2	125	2.10	2.48
A6	M/H	78.2	20.2	1.6	1970	79.2	1555	2726	29.9	174	< 0.1	2.86
A7	M/H	85.7	12.9	1.4	4829	102	1543	2643	17.9	211	< 0.1	2.29
Less than 53 μm size fraction												
A4	M/H				1133	94.7	1975	3042	41.5	124	< 0.1	3.38
A6	M/H				946	82.7	1910	2294	44.2	133	< 0.1	4.41
A7	M/H				1643	105	2547	3489	34.0	189	2.02	3.91
Bulk Soil[d]												
Rhyollitic (n = 7)					162	34.5	796	88	10.7	7.9	13.6	9.70
Melange (n = 16)					314	50.0	855	259	10.7	37.3	8.17	13.2
Serpentine (n = 10)					2373	87.1	1511	2929	4.4	150	3.61	1.69

[a] L = Low severity, M = Moderate severity, H = High severity
[b] Determined by sieve analysis
[c] Determined by laser diffraction particle size counter
[d] Bulk soil concentrations are mean values using all soil cores (fire-affected and unburned) for each geology type: rhyolitic (*n* = 7), mélange (*n* = 16), and serpentine (*n* = 10).

Supplementary Table 7: Results (W statistic and p-values) from Shapiro-Wilk tests to assess data normality for reactive Cr(VI) concentrations (μg kg^{-1}). Bold, italicized p-values indicate data do not meet normality assumption and thus were analyzed using nonparametric tests. The number of soil core replicates used to calculate means and standard errors (SE) for each soil type (same for surface and control depths) is reported as *n* and detailed in Table S1.

			Near Surface (0-2 cm)				Control Depth (10-16 cm)			
Geology	Fire Severity	*n*	Mean (μg kg^{-1})	± SE (μg kg^{-1})	W	p-value	Mean (μg kg^{-1})	± SE (μg kg^{-1})	W	p-value
Fire-Affected Sites										
Serpentinite	Low	3	73.0	16.5	0.780	0.068	18.5	2.9	0.985	0.768
Serpentinite	Mod/High	4	226.3	10.5	0.785	0.078	63.1	27.3	0.903	0.444
Serpentinite	All	7	160.6	32.1	0.841	0.100	44.0	17.2	0.725	***0.0069***
Rhyolite	Low	4	12.2	4.1	0.802	0.107	*nd*	-	-	-
Mélange	Low	9	40.7	10.4	0.889	0.196	11.0	3.2	0.770	***0.0093***
Unburned Sites										
Serpentinite	-	3	35.1	15.9	0.991	0.820	16.5	8.1	0.750	***0.000***
Rhyolite	-	3	*nd*	-	-	-	*nd*	-	-	-
Mélange	-	7	25.2	3.9	0.862	0.156	10.7	3.8	0.649	***0.0010***

Supplementary References

1. McClain, C. N., Fendorf, S., Webb, S. M. & Maher, K. Quantifying Cr(VI) Production and Export from Serpentine Soil of the California Coast Range. *Environ. Sci. Technol.* **51**, 141–149 (2017).
