# Supplementary material for: Metal toxin threat in wildland fires determined by geology and fire severity
Source: Nat Commun. 2023 Dec 12;14:8007. doi: 10.1038/s41467-023-43101-9 (PMC10716285; doi:10.1038/s41467-023-43101-9)
Supplement: Supplementary file 3 — Reporting Summary [file 41467_2023_43101_MOESM3_ESM.pdf]

nature portfolio

Corresponding author(s): Scott Fendorf

Last updated by author(s): Oct 9, 2023

Reporting Summary

Nature Portfolio wishes to improve the reproducibility of the work that we publish. This form provides structure for consistency and transparency in reporting. For further information on Nature Portfolio policies, see our Editorial Policies and the Editorial Policy Checklist.

Statistics

For all statistical analyses, confirm that the following items are present in the figure legend, table legend, main text, or Methods section.

n/a	Confirmed	
☐	☒	The exact sample size (*n*) for each experimental group/condition, given as a discrete number and unit of measurement
☐	☒	A statement on whether measurements were taken from distinct samples or whether the same sample was measured repeatedly
☐	☒	The statistical test(s) used AND whether they are one- or two-sided<br>*Only common tests should be described solely by name; describe more complex techniques in the Methods section.*
☐	☒	A description of all covariates tested
☐	☒	A description of any assumptions or corrections, such as tests of normality and adjustment for multiple comparisons
☐	☒	A full description of the statistical parameters including central tendency (e.g. means) or other basic estimates (e.g. regression coefficient) AND variation (e.g. standard deviation) or associated estimates of uncertainty (e.g. confidence intervals)
☐	☒	For null hypothesis testing, the test statistic (e.g. *F*, *t*, *r*) with confidence intervals, effect sizes, degrees of freedom and *P* value noted<br>*Give P values as exact values whenever suitable.*
☒	☐	For Bayesian analysis, information on the choice of priors and Markov chain Monte Carlo settings
☒	☐	For hierarchical and complex designs, identification of the appropriate level for tests and full reporting of outcomes
☒	☐	Estimates of effect sizes (e.g. Cohen's *d*, Pearson's *r*), indicating how they were calculated

Our web collection on statistics for biologists contains articles on many of the points above.

Software and code

Policy information about availability of computer code

Data collection	Coulter LS-230<br>Spectro XEPOS<br>Rigaku MiniFlex 600 with PDXL 2 software<br>Shimadzu UV-1601<br>Thermo Scientific XSERIES 2<br>Westco SmartChem 200 Discrete Analyzer
Data analysis	Microsoft Excel 2016<br>R (v. 4.1.3) ; stats package (v.4.1.3)<br>QGIS (v. 3.10.4)<br>Rigaku PDXL 2 software<br>Microanalysis Toolkit package (v. 2.04)<br>ATHENA software package (v. 0.9.26)

For manuscripts utilizing custom algorithms or software that are central to the research but not yet described in published literature, software must be made available to editors and reviewers. We strongly encourage code deposition in a community repository (e.g. GitHub). See the Nature Portfolio guidelines for submitting code & software for further information.

Data

Policy information about availability of data

All manuscripts must include a data availability statement. This statement should provide the following information, where applicable:

- Accession codes, unique identifiers, or web links for publicly available datasets
- A description of any restrictions on data availability
- For clinical datasets or third party data, please ensure that the statement adheres to our policy

Data supporting the findings of this study are available within the article, its Supplementary Information file, and source data have been deposited in the Stanford Digital Repository at https://purl.stanford.edu/gj966nd5234. Geospatial data are publicly available online. California regional geologic maps are available at the United States Geological Survey (USGS) (https://pubs.er.usgs.gov). Vegetation data for Sonoma County are available at https://sonomavegmap.org. California soil data are available at https://websoilsurvey.nrcs.usda.gov. Global fire perimeters (2001-2020) are available at the Global Wildfire Information System (GWIS) (https://gwis.jrc.ec.europa.eu). A generalized global geologic map is available at https://geoscan.nrcan.gc.ca. Global population projection grids are available at NASA Socioeconomic Data and Applications Center (SEDAC) (https://beta.sedac.ciesin.columbia.edu).

Human research participants

Policy information about studies involving human research participants and Sex and Gender in Research.

Reporting on sex and gender	*Use the terms sex (biological attribute) and gender (shaped by social and cultural circumstances) carefully in order to avoid confusing both terms. Indicate if findings apply to only one sex or gender; describe whether sex and gender were considered in study design whether sex and/or gender was determined based on self-reporting or assigned and methods used. Provide in the source data disaggregated sex and gender data where this information has been collected, and consent has been obtained for sharing of individual-level data; provide overall numbers in this Reporting Summary. Please state if this information has not been collected. Report sex- and gender-based analyses where performed, justify reasons for lack of sex- and gender-based analysis.*
Population characteristics	*Describe the covariate-relevant population characteristics of the human research participants (e.g. age, genotypic information, past and current diagnosis and treatment categories). If you filled out the behavioural & social sciences study design questions and have nothing to add here, write "See above."*
Recruitment	*Describe how participants were recruited. Outline any potential self-selection bias or other biases that may be present and how these are likely to impact results.*
Ethics oversight	*Identify the organization(s) that approved the study protocol.*

Note that full information on the approval of the study protocol must also be provided in the manuscript.

Field-specific reporting

Please select the one below that is the best fit for your research. If you are not sure, read the appropriate sections before making your selection.

☐ Life sciences ☐ Behavioural & social sciences ☒ Ecological, evolutionary & environmental sciences

For a reference copy of the document with all sections, see nature.com/documents/nr-reporting-summary-flat.pdf

Ecological, evolutionary & environmental sciences study design

All studies must disclose on these points even when the disclosure is negative.

Study description	In this study, we examined the combination of soil properties and fire severity governing Cr(VI) formation within fire-impacted landscapes, with a particular focus on Cr(VI) presence in wind-dispersible particulates. We analyzed reactive Cr(VI) concentrations in burned and unburned soils collected as intact cores and from bulk surface soil and ash from four ecological preserves that burned during the 2019 Kincade Fire or the 2020 LNU Lightning Complex in the North Coast Range, California, USA. These preserves provided variation in ecosystems (grassland, chaparral, and forest), a gradient in burn severity, and contrasting geologies (felsic to ultramafic, or metamorphic equivalents) with highly variable soil Cr concentrations. Post-fire Cr(VI) concentrations approximately one month and a year after fire were compared to assess the environmental persistence of the toxin.
Research sample	Soil (0-30 cm) and the overlying surface layer of disaggregated soil and ash particles.
Sampling strategy	For each environmental covariate (geology, fire severity), we collected at least three soil cores in the field. Post-fire environmental hazards, such as burned trees and infrastructure, limited access to some planned sampling sites, dictate aspect of sampling.<br>Near surface soil samples were collected from fire-affected and unburned locations as intact cores using a slide hammer soil core sampler and cased in plastic liners with caps. Soil cores ranged from 15-30 cm and depended on the refusal depth at each sampling location. A total of 38 cores were collected across the four preserves, 22 from burned areas and 16 from unburned locations. Among all sampling locations, 19 cores were collected from grasslands, 10 cores were from chaparral, and 9 cores from mixed forests. Ten

	cores were from serpentine (ultramafic) soil, 7 from rhyolitic (felsic) soil, 16 soil cores were from mélange (having similar chemistry as mafic rocks) parent material, and 5 were from sandstone (similar chemistry as mafic). Within a serpentine chaparral, 4 cores were characterized as moderate-high burn severity, 3 cores were low burn severity, and 3 cores were designated as unburned.
Data collection	The authors collected all primary data for the project using the instruments and software listed in the Software and Code - Data Collection section. Data collected from Thermo Scientific XSERIES 2 was acquired and initially prepared by instrument facility manager, Dr. Guangchao Li. Dr. Juan Lezama Pacheco assisted Dr. Lopez in collecting and analyzing solid-phase data using Rigaku MiniFlex 600 and at the Stanford Synchrotron Radiation Lightsource.
Timing and spatial scale	Fieldwork was completed in November 2019, September 2020, and July 2021. Prior to analysis, field samples were stored in the refrigerator in airtight containers to minimize changes in chemical conditions. Sample preparation and aqueous extractions began in January 2020, but was stopped in March 2020 due to COVID-19 shutdown. Laboratory access was prohibited until June 2020, when sample analysis resumed. Samples analyzed in January 2020 were re-extracted to confirm minimal chemical changes in storage.
Data exclusions	Some soil depth intervals, particularly in near surface depths, did not have sufficient mass for triplicate extractions, and thus, one or two replicates were completed. For reactive Cr(VI) measurements, these soil cores were: F6 (0-1cm), F1 (3-4cm), M15 (1-2cm), M11 (10-12cm), M8 (0-1cm), M2 (5-6cm), M2 (0-1cm, 1-2 cm). Due to limited fire impacts below 6 cm, we did not analyze ammonium concentrations in soil depths below 6 cm, and replicates were further limited by remaining aqueous volume post-extraction and Cr (VI) analysis.
Reproducibility	Aqueous analyses were completed in triplicate (individual extractions) to assess sample variability. Extractions for a subset of soil and ash samples were repeated months later (after refrigerator storage in airtight containers) and at different extraction times to confirm similar aqueous concentrations and limited sample reactivity. We also compared unburned soil concentrations with measurements collected by a different research team at a proximal location near the serpentine chaparral sampled in this study.
Randomization	Soil samples were allocated into groups based on geologic substrate, ecosystem type (vegetation class), and fire severity (unburned, low, moderate/high). Within each soil core, soil samples were also grouped based on depth. Specifically, the top 6-cm of the soil cores were manually divided into 1-cm intervals and the remaining depths were sliced in 2-cm intervals to an analysis depth of 16-cm.
Blinding	Soil and ash samples were not blinded during this study. Soil and ash color and coarse materials removed by sieving were associated with geologic substrate and fire severity, and these observations could not be blinded.

Did the study involve field work? ☒ Yes ☐ No

Field work, collection and transport

Field conditions	The three preserves (Modini, White Rock, and Pepperwood Preserve) that burned during the Kincade Fire were sampled twice, once immediately following the fire's complete containment (October 23-November 6, 2019) on November 15th and 19th, 2019, and then approximately 11 months post-fire on September 18th, 2020. Both sampling days in November 2019 were clear weather and 18-20 degrees C, and September 18th, 2020, was clear weather and a high temperature of 24 degrees C. Sampling sites at McLaughlin Natural Reserve were sampled once on September 19th, 2020, under similar weather conditions before full containment of the LNU Lightning Complex (August 17-October 2, 2020), and bulk surface soil and ash were resampled on July 29th, 2021.
Location	Sonoma, Napa, and Lake County in California, USA, at four natural preserves: Pepperwood Preserve (38.5698°, -122.6917°, approximately 200-475 m elevation, 1295 hectares), White Rock Preserve (38.7082°, -122.8332°, approximately 150-300 m elevation, 37 hectares), Modini Preserve (38.7069°, -122.7625°, approximately 300-770 m elevation, 1214 hectares), McLaughlin Natural Reserve (38.8712°, -122.4215°, approximately 650 m elevation, 2833 hectares)
Access & import/export	Research Use and Access Applications: In order to access preserves for post-fire field sampling, we reached out to managers at each preserve and completed required research use applications that were internally reviewed and approved by advisory committees.<br>Pepperwood Preserve - November 2019<br>Sonoma Land Trust (access to White Rock Preserve) - November 2019<br>Audubon Canyon Ranch (access to Modini Preserve) - November 2019<br>University of California Natural Reserve System (access to McLaughlin Natural Reserve) - September 2020<br><br>2020 COVID Fieldwork Access:<br>Additional application was approved by Stanford University and approvals from each preserve/organization to conduct field research in compliance with local COVID-19 ordinances.
Disturbance	We worked with Preserve Managers to minimize environmental impacts. Soil and ash samples were collected either as intact 30-cm cores using a slide hammer or in bulk ziploc bags with a shovel or trowel. Disturbance was minimized by refilling holes created in the ground with surrounding soil. We also cleaned all equipment and gear between preserves to avoid introducing invasive species. Lastly, while walking across landscapes, we avoided vegetation that survived wildfires.<br><br>For our research question, immediate post-fire soil and ash sampling was completed before any precipitation events to minimize Cr (VI) transport and prevent blurring of fire effects on the physicochemical composition within the top 30-cm of soil profiles.

Reporting for specific materials, systems and methods

We require information from authors about some types of materials, experimental systems and methods used in many studies. Here, indicate whether each material, system or method listed is relevant to your study. If you are not sure if a list item applies to your research, read the appropriate section before selecting a response.

Materials & experimental systems

n/a	Involved in the study	
☒	☐	Antibodies
☒	☐	Eukaryotic cell lines
☒	☐	Palaeontology and archaeology
☒	☐	Animals and other organisms
☒	☐	Clinical data
☒	☐	Dual use research of concern

Methods

n/a	Involved in the study	
☒	☐	ChIP-seq
☒	☐	Flow cytometry
☒	☐	MRI-based neuroimaging
